# Simulating Ideal Assistive Devices to Reduce the Metabolic Cost of Running

**DOI:** 10.1371/journal.pone.0163417

**Published:** 2016-09-22

**Authors:** Thomas K. Uchida, Ajay Seth, Soha Pouya, Christopher L. Dembia, Jennifer L. Hicks, Scott L. Delp

**Affiliations:** 1 Department of Bioengineering, Stanford University, Stanford, California, United States of America; 2 Department of Mechanical Engineering, Stanford University, Stanford, California, United States of America; 3 Department of Orthopaedic Surgery, Stanford University, Stanford, California, United States of America; Norwegian University of Science and Technology, NORWAY

## Abstract

Tools have been used for millions of years to augment the capabilities of the human body, allowing us to accomplish tasks that would otherwise be difficult or impossible. Powered exoskeletons and other assistive devices are sophisticated modern tools that have restored bipedal locomotion in individuals with paraplegia and have endowed unimpaired individuals with superhuman strength. Despite these successes, designing assistive devices that reduce energy consumption during running remains a substantial challenge, in part because these devices disrupt the dynamics of a complex, finely tuned biological system. Furthermore, designers have hitherto relied primarily on experiments, which cannot report muscle-level energy consumption and are fraught with practical challenges. In this study, we use OpenSim to generate muscle-driven simulations of 10 human subjects running at 2 and 5 m/s. We then add ideal, massless assistive devices to our simulations and examine the predicted changes in muscle recruitment patterns and metabolic power consumption. Our simulations suggest that an assistive device should not necessarily apply the net joint moment generated by muscles during unassisted running, and an assistive device can reduce the activity of muscles that do not cross the assisted joint. Our results corroborate and suggest biomechanical explanations for similar effects observed by experimentalists, and can be used to form hypotheses for future experimental studies. The models, simulations, and software used in this study are freely available at simtk.org and can provide insight into assistive device design that complements experimental approaches.

## Introduction

Designing assistance for running vast distance is now quite a common pursuit, As reducing the power your muscles devour could markedly ease your commute. A current concern is how to discern what torque would be best at each joint—We use simulations to search for locations and patterns of torques to appoint. The pipeline we use could help someone to choose an assistive device to design, With the energy burned by each muscle concerned guiding how to revise and refine.

T. K. U.

The human body has evolved over millions of years into a system that is efficient at bipedal locomotion [1] while remaining amazingly versatile. A consequence of this versatility is that the properties of our musculoskeletal system are not ideally suited for any single physical activity in which we engage. An analogous situation is readily apparent in penguins, which are expert swimmers but, despite their formal attire, move about rather inelegantly on land. Indeed, the waddling of penguins is both slower and more energetically expensive than the terrestrial locomotion observed in other bipedal birds [2]. Humans, though perhaps more adept at running than are penguins at walking, nevertheless have a morphology that also represents a compromise between different forms of locomotion [3] with different mechanical and energetic properties [4]. For example, efficient walking requires more compliant tendons than efficient running [5]. Cyclists shift gears to maintain a comfortable pedaling frequency (and, in turn, favorable muscle fiber velocities) as ground speed varies [6]. Because our muscles and tendons cannot instantaneously “shift gears” when we transition between gaits or change speed, their properties must represent a balance among competing demands in different movement scenarios. This compromise provides a possible explanation for why running economy is insensitive to speed [7], acceleration/deceleration cycles [8], and footstrike pattern [9].

There are many natural means by which the properties of our muscles and tendons can change. For example, muscle fibers decrease in strength and contraction speed as we age [10], and tendon compliance can be affected by strength training [11]. Although proper training can improve running efficiency over time, simply donning an assistive device would offer several advantages. First, training requires prolonged effort with changes occurring relatively slowly. Secondly, the dynamic properties of muscles and tendons are fundamentally limited by the physical properties of their constituent tissues; the dynamic properties of assistive devices are not bound by these biological constraints. Finally, our bodies will always compromise between competing demands, such as maximizing performance while retaining some amount of versatility in our movement. Assistive devices can overcome these challenges, allowing us to instantaneously modify the dynamics of our musculoskeletal system, temporarily sacrificing versatility to maximize performance at a specific task.

### Progress in Assistive Device Design

The earliest designs of exoskeleton-like devices were conceived in the late 1800s to assist walking, running, and jumping [12]. In the 1960s, General Electric and the United States Department of Defense made the first attempt at building a practical powered exoskeleton for lifting heavy loads, though it was too heavy, bulky, unstable, and energetically inefficient to be practical [13]. The first functional, untethered (i.e., energetically autonomous) exoskeleton for carrying heavy loads was developed at Berkeley in the early 2000s [14]. Potential applications of this technology include providing load-carriage assistance to military and emergency personnel to prevent injury and increase endurance. Similar technologies have already been applied to facilitate physical therapy [15], to help restore bipedal locomotion to individuals with paraplegia [16], and to reduce muscle fatigue in unimpaired and elderly individuals [17, 18]. Thorough reviews on the development of exoskeletons and assistive devices are available in the literature [19–21].

A central objective of assistive device design is to reduce the energy expended by the wearer. This goal was accomplished for unloaded walking in 2013 by Malcolm et al. [22] using a tethered device that provides an assistive ankle plantarflexion moment during push-off. Malcolm et al. tested several actuation profiles and observed the greatest metabolic cost reduction of 6±2% when the actuator torque had a later onset time and lower magnitude than the total moment generated by the plantarflexor muscles during unassisted walking. The following year, Mooney et al. [23] reported metabolic cost reductions of 8±3% during loaded walking, again providing an ankle plantarflexion moment during push-off, but using an untethered device. Mooney et al. noted the importance of minimizing the mass of the device, as mass added to the leg has increasingly detrimental effects on metabolic cost as its location moves distally [24]. In 2015, Collins et al. [25] reported metabolic cost reductions of 7.2±2.6% during unloaded walking using an untethered and unpowered ankle plantarflexion device, leading to the provocative suggestion that the structure of the human body could, in theory, further evolve to be more energetically efficient during walking.

### Current Challenges in Assistive Device Design

These impressive advancements in walking assistance have yet to be repeated for running. Several studies have used assisted hopping to isolate the bouncing component of the running motion, where the legs act like compressive springs during ground contact [26]. Grabowski and Herr [27] reported metabolic savings of up to 28% during hopping using leaf springs spanning the ankle, knee, and hip. Farris and Sawicki [28] reported metabolic savings of 12% using a passive spring-loaded ankle exoskeleton and noted the importance of tuning the exoskeleton parameters for each subject. Farris and Sawicki also highlighted the potential benefits of assisting muscles crossing the knee, given that a substantial proportion of the total positive power was generated by the knee extensors. In 2008, Dollar and Herr [29] described the first device designed specifically to assist running: an energetically autonomous knee brace that places a spring in parallel with the knee during stance and allows the knee to bend freely during swing. The intention was to store and release energy that would otherwise be absorbed by the quadriceps; however, a reduction in metabolic cost was never reported for this device. Cherry et al. [30] designed an exoskeleton incorporating a carbon composite leaf spring spanning the ankle, knee, and hip, but reported challenges due to device inertia. In 2015, Sugar et al. [31] presented devices that inject power at precise times during cyclic tasks like hopping and running, and reported a reduction in metabolic cost of up to 10.2% in a “tall male” subject (mass 66.1 kg, height 1.82 m) by applying hip flexion and extension moments during running—though the metabolic cost reported for a “small female” subject (mass 59.1 kg, height 1.62 m) increased by 6.7% when using the same device.

Device designers face several challenges. First, devices are being developed for a biological system that is complex, already finely tuned, and neither fully characterized nor fully understood. Thus, it is not immediately apparent how to provide assistance, nor is it straightforward to predict how subjects will adapt to a particular device. For example, Lenzi et al. [32] developed an exoskeleton that applied an assistive flexion/extension torque at the hip during walking and found the most significant reductions in the activity of the rectus femoris and the gastrocnemius, which was surprising given that the gastrocnemius crosses only the ankle and knee. There are also challenges related specifically to designing devices using primarily experimental studies. Physical testing requires the design and fabrication of prototypes, which can be time-consuming and expensive, and may require several design iterations to refine key device parameters [33]. A central requirement is to minimize the mass added to the leg [23, 34], particularly of the components attached most distally [24]. Minimizing distal mass can require special accommodation at the design stage (e.g., by mounting a spring in a backpack and transmitting force to the knee using a Bowden cable [30]) and can increase the cost of components (e.g., by fabricating selectively reinforced carbon fiber frames for each subject [25]). Experimentalists circumvent this problem by tethering the device to offboard actuators and power supplies [22, 35], but experiments may then be limited to synthetic, lab-based scenarios. Human subject testing often introduces additional obstacles, such as obtaining ethics approval, recruiting (exo)suitable participants, guaranteeing subject safety, building subject-specific prototypes [25], and collecting as much data as possible in a limited timeframe using only noninvasive sensors. Finally, physical prototyping can introduce confounding effects, such as compliant attachments to the body [36], variability within and between subjects [31, 37], and training effects [38, 39].

### Simulation-based Design of Devices to Assist Running

We advocate simulation-based design as a tool to overcome many of the challenges facing device designers. The automotive, aerospace, and other industries have used virtual prototyping for decades to complement experimental design approaches, reducing development time and cost. Simulation-based design reduces the need to build physical prototypes and enables fast, automated, and repeatable testing in completely controlled virtual environments where hazardous scenarios can be studied without risk of injury. Simulations can also be used to probe complex systems in ways that are otherwise impossible. For example, our simulations of the human musculoskeletal system allow us to study the recruitment and energetics of a subject’s individual muscles—even deep muscles—in a completely noninvasive way [40–43]. We are also able to isolate design elements of assistive devices that are difficult to isolate experimentally, such as the effects of added mass, actuator limitations, frictional losses, compliant attachments to the body, and kinematic adaptations [44–46].

In this work, we addressed a critical first step in adoption of simulation-based assistive device design by adding ideal assistive devices to the lower limb in simulations of running, and seeking insight into the biomechanical and energetic effects of the simulated devices. Each assistive device was modeled as a massless, lossless actuator that applied a torque directly to the joint, where neither the magnitude nor the rate of assistive torque was limited. We performed muscle-driven simulations of 10 subjects running at 2 and 5 m/s using several combinations of ideal assistive devices, and computed the average metabolic power consumed by each muscle in each scenario. We compared reductions in average metabolic power across running speeds and assistance strategies, and studied changes in the recruitment, energetics, and dynamics of individual muscles. We used our simulations to test three hypotheses: (i) a particular assistance location may be more effective at one speed than another, as reported when assisting the hip during running [31]; (ii) the ideal assistive torque differs in magnitude and timing from the total joint moment generated during unassisted running, as suggested experimentally when assisting the ankle during walking [22]; and (iii) a device can decrease activity in muscles that do not cross the assisted joint, as observed when assisting the hip during walking [32].

## Methods

We generated simulations of 10 male long-distance runners using the data, models, and methods reported by Hamner and Delp [42]. The Stanford University Institutional Review Board approved the experimental protocol and subjects provided informed written consent. We used a three-dimensional musculoskeletal model with 29 degrees of freedom, 92 lower extremity and torso muscles, and arms driven by torque actuators, which has been used previously to study how each muscle contributes to accelerating the body’s center of mass during running [42, 47]. Each lower limb in the model has five degrees of freedom in our simulations: hip flexion/extension, hip abduction/adduction, hip internal/external rotation, knee flexion/extension, and ankle plantarflexion/dorsiflexion. We used the Computed Muscle Control (CMC) tool in OpenSim 3.2 [48–51] to generate muscle-driven simulations of running at 2 and 5 m/s (13.4 and 5.4 min/mi; 8.3 and 3.3 min/km) for each subject. The energy consumed by each muscle was computed from the CMC simulation results using a modified version of the muscle energetics model proposed by Umberger et al. [52]; our modifications to this model have been described previously and have been validated for running over the range of speeds studied here [43]. The muscle energetics model used in this study is available in OpenSim 3.3 and as a plug-in for OpenSim 3.2.

We investigated the effect of adding (i) ideal flexion/extension devices bilaterally to the ankle, knee, and hip separately and in all combinations, and (ii) ideal hip flexion/extension devices with hip abduction/adduction and internal-/external-rotation assistance. We first simulated three running gait cycles for each subject at each speed, following the methods described by Hamner and Delp [42]. We then added ideal assistive devices to the ankle, knee, and/or hip joints by increasing the strength of the corresponding “reserve actuators” in our OpenSim model, and repeated each simulation while applying the original ground reaction forces and tracking the original kinematics. The reserve actuators ordinarily apply small joint torques directly to the skeleton that compensate for muscle weaknesses in the model, should any be encountered during the simulation. CMC balances the recruitment of reserve actuators with that of the muscles by minimizing the following instantaneous objective function:

$$J\left(a,\tau \right)=\sum_{i=1}^{\text{nMuscles}}a_{i}^{2}+\sum_{j=1}^{\text{nReserves}}{\left(\frac{\tau_{j}}{w_{j}}\right)}^{2},$$ (1)

where nMuscles and nReserves are the number of musculotendon and reserve actuators in the model, *a*_*i*_ ∈ [0.02, 1] is the instantaneous activation of the *i*th muscle, *τ*_*j*_ is the instantaneous torque applied by the *j*th reserve actuator, and *w*_*j*_ is a constant weighting factor (the “optimal force” property in OpenSim) that scales the penalty associated with recruiting the *j*th reserve actuator. To simulate ideal actuators, we modified the values of the corresponding weighting factors *w*_*j*_, replacing their original values of 1 N·m with 1 MN·m. We then used CMC to determine the muscle activations and actuator torques that would minimize the objective function *J*. As shown in Eq (1), a weighting factor of 1 N·m penalizes the solution by the square of the torque generated by the corresponding reserve actuator; as such, the peak reserve actuator torques were very small in the original simulations (less than 0.05 N·m/kg [42]). A weighting factor of 1 MN·m results in a negligible penalty when the corresponding reserve actuator is recruited (the square of one millionth of the generated torque), thus predicting the torque that would be applied by an ideal assistive device. Note that the net joint moments were the same regardless of the value of *w*_*j*_ because the same ground reaction forces were applied and the same kinematics were tracked. We did not add an assumed device mass to the model, nor did we limit the magnitude or rate of the assistive torque. In total, we performed 660 simulations in this study.

We used our muscle energetics model to predict the instantaneous metabolic power consumed by each lower extremity muscle for each subject, speed, and assistance scenario. We calculated the average metabolic power consumption for each muscle by integrating its instantaneous power consumption over the gait cycle (thereby computing the total metabolic energy consumed in one gait cycle) and dividing by the cycle duration. We then summed over all muscles and divided by the mass of the subject to obtain the total average metabolic power consumption (in W/kg). These calculations were repeated for and averaged over three running gait cycles for each subject, speed, and assistance scenario. We subtracted the average metabolic power consumed when unassisted from that consumed in each assistance scenario to measure the performance of each ideal assistive device; the mean and standard deviation across all subjects were then calculated at each speed and in each assistance scenario. Because physical devices often apply assistance unidirectionally (e.g., assisting ankle plantarflexion but not dorsiflexion), we also approximated the savings in metabolic power attributable to ankle, knee, and hip devices that assist only flexion or only extension. We obtained these approximations by first partitioning each simulation into segments during which the ankle, knee, or hip flexion/extension actuator was generating only flexion or only extension torque; we then summed the reductions in metabolic power associated with flexion and extension assistance separately.

The metabolic power consumed by each muscle was ascribed to each functional group (e.g., ankle plantarflexion) based on the proportion of mechanical power the muscle generated in each degree of freedom. The metabolic power consumed by a muscle crossing a single one-degree-of-freedom joint (e.g., the soleus in our model) was trivially ascribed entirely to the only functional group to which it can contribute. For each of the remaining muscles and at each time step of the simulation, we first summed the magnitudes of the mechanical power generated at each degree of freedom. The muscle’s metabolic power consumption at each instant of time was then apportioned to each functional group according to the proportion of the corresponding mechanical power relative to this sum. For example, if the ankle and knee had equal angular velocities at a particular instant of time and the gastrocnemius had moment arms of 5.0 and 2.5 cm at the ankle and knee, respectively, then two-thirds of its metabolic power consumption at that time would be ascribed to ankle plantarflexion and one-third would be ascribed to knee flexion. If, instead, the angular velocity of the ankle were zero, then all the metabolic power consumed by the gastrocnemius at that instant of time would be ascribed to knee flexion. We summed the contributions of all muscles to each functional group to obtain an estimate of the metabolic power consumed in order to perform ankle plantarflexion and dorsiflexion, knee flexion and extension, and hip flexion, extension, abduction, adduction, internal rotation, and external rotation.

## Results and Discussion

**Fig 1:**
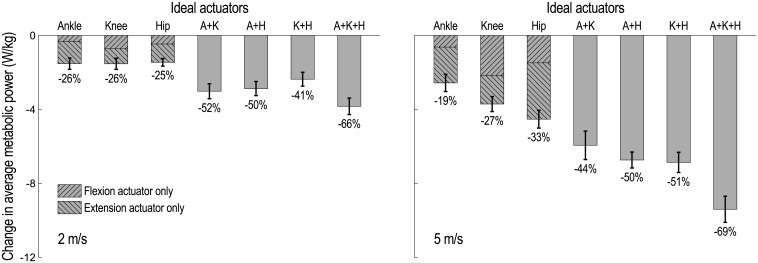
Change in average metabolic power consumed by lower extremity muscles when running with ideal flexion/extension assistive devices. Flexion/extension actuators were added bilaterally at the ankle (A), knee (K), and/or hip (H). The mean (column) and standard deviation (vertical line) over 10 subjects are shown for seven assistance scenarios when running at 2 m/s (left) and 5 m/s (right). The hatched regions approximate the change in average metabolic power attributable to unidirectional (i.e., only flexion or only extension) assistive torques. Change in metabolic cost is reported as power averaged over the gait cycle and normalized by subject mass (vertical axis), and as a percentage (indicated below each column); both quantities are expressed relative to the average metabolic power consumed in the unassisted simulations at each speed. When running at 2 m/s, the three actuators were approximately equally effective when used separately; when running at 5 m/s, the hip actuator was significantly more effective than the ankle or knee actuators (*p* < 0.002, matched pairs t-test).

Average metabolic power consumption decreased in all assistance scenarios and at both running speeds (Fig 1). Reductions in metabolic power were greater in magnitude when running at 5 m/s, but were similar between speeds when expressed as a percentage of the metabolic power consumed when running without assistance (indicated below each column in Fig 1). When running at 2 m/s, the ankle, knee, and hip flexion/extension actuators were approximately equally effective when used separately, reducing average metabolic power by 1.5 W/kg or about one-quarter of the metabolic power consumed when unassisted; at 5 m/s, however, the hip actuator saved significantly more metabolic power than the ankle or knee actuators (*p* < 0.002, matched pairs t-test). The average metabolic power saved when assisting more than one joint was less than or equal to the sum of the savings when assisting each joint separately. If unidirectional assistance (i.e., only flexion or only extension torques) were provided, extension torques would result in greater savings than flexion torques for the ankle and hip joints. If assisting the knee, providing only flexion torques would be marginally less beneficial than only extension torques when running at 2 m/s, but would be substantially more beneficial at 5 m/s.

**Fig 2:**
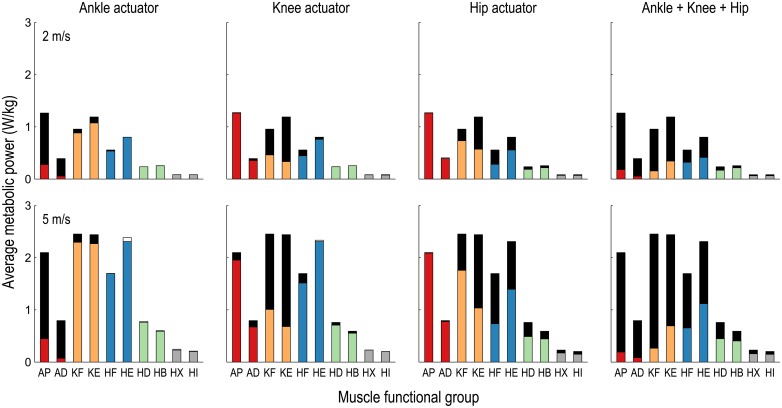
Average metabolic power consumed by lower extremity muscle groups when running with ideal flexion/extension assistive devices. The average metabolic power consumed over the running gait cycle, normalized by subject mass and averaged over 10 subjects, is shown for the ankle plantarflexors and dorsiflexors (AP and AD, red), the knee flexors and extensors (KF and KE, orange), and the hip flexors, extensors, adductors, abductors, external rotators, and internal rotators (HF and HE, blue; HD and HB, green; HX and HI, gray) when running at 2 m/s (top row) and 5 m/s (bottom row) in four assistance scenarios. The total height of each column, excluding the white regions, indicates the metabolic cost associated with the corresponding functional group in the unassisted simulation. Black and white regions indicate, respectively, reductions and increases in average metabolic power when the assistive devices were added. The greatest savings were observed in muscles actuating the assisted degrees of freedom, but substantial savings were observed in other muscle groups as well.

The greatest reductions in average metabolic power were observed in muscles actuating the assisted degrees of freedom, but substantial savings were observed in other muscle groups as well (Fig 2). When assisting ankle plantarflexion/dorsiflexion, the greatest reductions in metabolic power occurred in the ankle plantarflexors and dorsiflexors; only small reductions were observed in the knee flexors and extensors. In contrast, when assisted by ideal hip flexion/extension actuators, the reductions in metabolic power observed in the knee flexors and extensors were comparable to the reductions observed in the hip flexors and extensors. When running at 5 m/s, the average metabolic power attributable to knee extension decreased by 72% when assisting the knee and by 58% when assisting hip flexion/extension. Note that the knee extension cost decreased by more than half in each of these two scenarios, thus explaining why the average metabolic power saved when assisting the knee and hip simultaneously was less than the sum of the savings when assisting the knee and hip separately (Fig 1). The metabolic power attributed to flexing and extending the hip reduced by only about half when using ideal ankle, knee, and hip flexion/extension actuators simultaneously because the muscles crossing the hip remained responsible for generating hip abduction/adduction and internal/external rotation moments. Trends in the average metabolic power consumed by each lower extremity muscle group were similar at 2 and 5 m/s in each assistance scenario.

**Fig 3:**
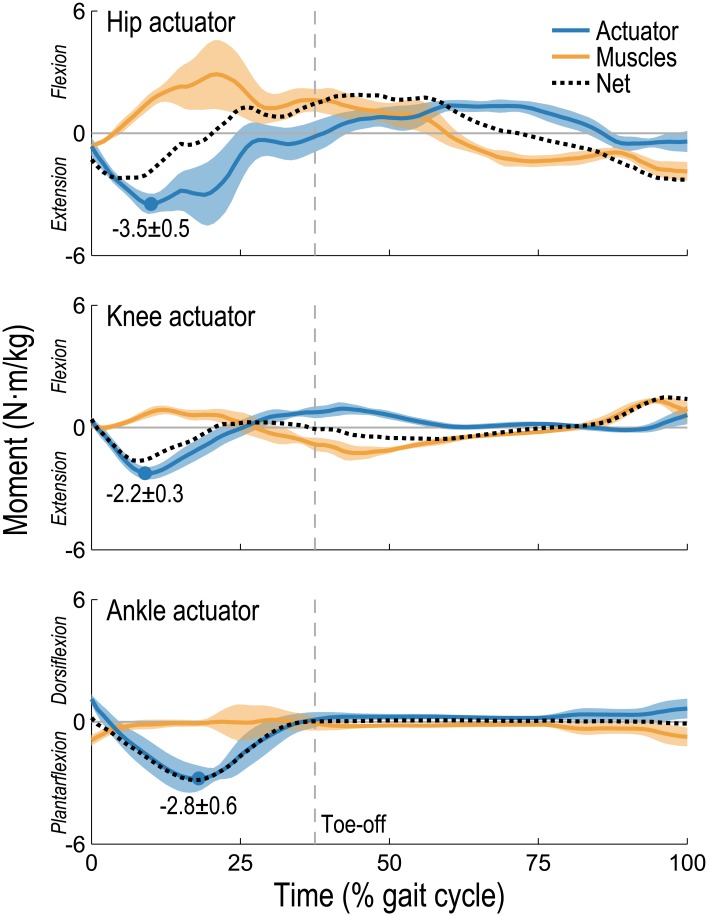
Actuator torque and total muscle moment acting about the same degree of freedom when assisting one joint. The actuator torque (blue), total muscle moment (orange), and net joint moment (black, dotted) for the right leg are shown normalized by subject mass over a running gait cycle (from foot-strike to foot-strike); the mean (line) and standard deviation (shaded region) across 10 subjects are shown when running at 5 m/s with hip (top), knee (center), or ankle (bottom) flexion/extension assistance. Circles indicate peak mean actuator torques; dashed vertical lines indicate the toe-off time (separating stance from swing), averaged across all subjects. The ideal actuator provided most of the joint moment in the ankle assistance scenario; however, when assisting the knee or hip, the total muscle moment was often opposing the actuator torque.

The assistance torques generated by the ideal actuators did not always resemble the corresponding net joint moments. When running at 5 m/s, the ideal ankle actuator generated roughly the entire net joint moment whereas the torques generated by the knee and hip actuators deviated substantially from their respective net joint moments (Fig 3). When assisting the knee or hip, the total muscle moment was often opposing the torque generated by the ideal actuator. For example, a large hip flexion moment was generated by the muscles during stance, which opposed a large extension torque generated by the ideal hip actuator. The magnitude of the peak mean actuator torque was greatest in the hip actuator (3.5 N·m/kg) and least in the knee actuator (2.2 N·m/kg). The standard deviation of the actuator torques and total muscle moments across subjects were substantially greater at the hip than at the knee or ankle, which may have important design implications (see the Hypothesis Testing section, below).

### Predicted Changes in Muscle Coordination

**Fig 4:**
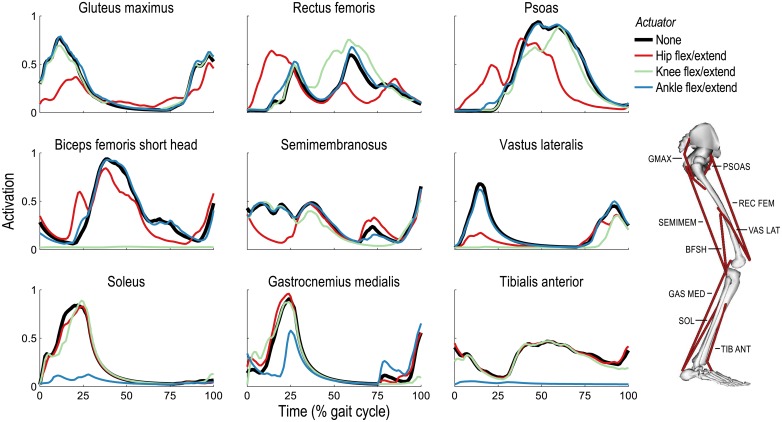
Activations of nine representative lower extremity muscles when running at 5 m/s and assisting one joint. Mean activations are shown for three uniarticular muscles on the posterior side of the right leg (left column), three uniarticular muscles on the anterior side of the leg (right column), and three biarticular muscles (center column) in four scenarios: unassisted (black) and when assisted by ideal hip (red), knee (green), or ankle (blue) flexion/extension actuators. Note that the vastus lateralis and rectus femoris insert into the patella (not shown in the diagram at right), thereby allowing them to generate knee extension moments. Ankle and knee actuators dramatically reduced activations of uniarticular muscles crossing the ankle and knee, respectively; the effect of the hip flexion/extension actuator was unique, in part, because the hip joint has two additional degrees of freedom. When knee or hip actuators were added, the rectus femoris activation increased in some parts of the gait cycle to take advantage of its relatively high force-generating capacity.

It is instructive to consider the changes in muscle activations when assistance is added because the metabolic power consumed by a muscle depends on its activity (other factors include the length, velocity, and composition of its muscle fibers [43]). When running at 5 m/s and assisting one joint in the sagittal plane, activations generally decreased in muscles crossing assisted joints; however, activations also decreased in muscles crossing *unassisted* joints, and some activations *increased* when assistance was added (Fig 4). When assisting the ankle, the only substantial changes in activations occurred in muscles crossing the ankle. The soleus and tibialis anterior activations decreased dramatically throughout the gait cycle because these muscles can generate only ankle moments; the ideal actuator generated these moments at negligible cost (see Eq (1)) and did so without affecting the muscle moments generated at the knee or hip. The activation of the gastrocnemius medialis decreased only partially during stance: although it was no longer responsible for generating an ankle plantarflexion moment, it was still recruited to generate a flexion moment at the (unassisted) knee.

**Fig 5:**
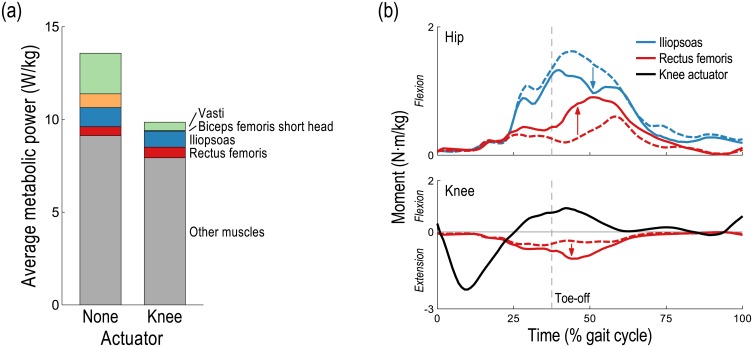
Effect of knee assistive device on energetics and dynamics of key muscles when running at 5 m/s. (a) The average metabolic power consumed by the two muscles whose energy consumption decreased the most (vasti and biceps femoris short head) and by the iliopsoas and rectus femoris, whose energy consumption decreased and increased, respectively. (b) Mean hip (top) and knee (bottom) flexion moments are shown for the right leg when unassisted (dashed lines) and when assisted by an ideal knee actuator (solid lines), averaged across 10 subjects; the mean knee actuator torque from Fig 3 is shown for reference (black). The rectus femoris had a higher force-generating capacity than the iliopsoas during early swing because the rectus femoris muscle fibers were lengthening while those of the iliopsoas were shortening. Thus, the recruitment of the rectus femoris increased to generate more of the necessary hip flexion moment, and the superfluous knee extension moment it generated was neutralized by the ideal actuator.

When assisting the knee, substantial changes in activations occurred in muscles crossing the knee and/or hip (Fig 4). The activations of the biceps femoris short head and vastus lateralis (two uniarticular muscles crossing the knee) decreased dramatically because the knee moments they generated when unassisted could be generated by the ideal actuator at negligible cost and without affecting the moments generated at the other joints. The activation of the rectus femoris increased during early swing to take advantage of its relatively high force-generating capacity. A muscle fiber can generate more force when lengthening than when shortening [53] and the rectus femoris muscle fibers were lengthening during early swing while those of the iliopsoas (the iliacus and psoas, two uniarticular hip flexors in our model) were shortening. The increased hip flexion moment generated by the rectus femoris enabled a decrease in the activity of the iliopsoas; the increased knee extension moment generated by the rectus femoris was neutralized by the ideal actuator at negligible cost (Fig 5(b)).

**Fig 6:**
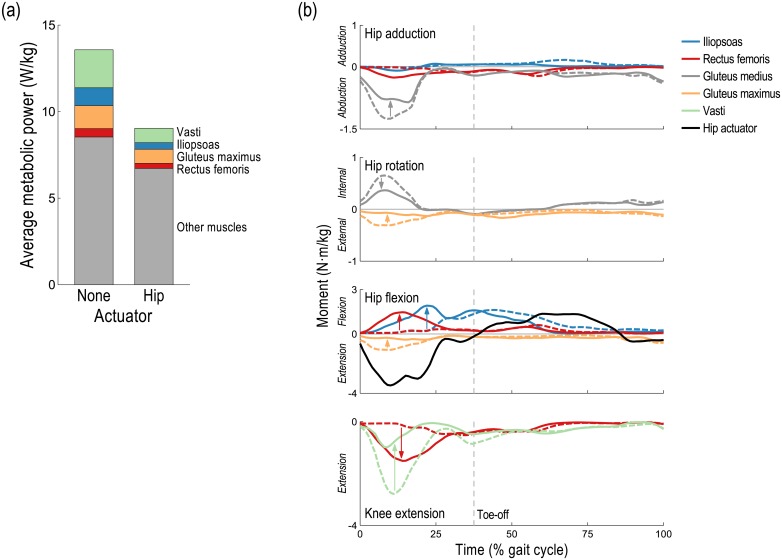
Effect of hip flexion/extension device on energetics and dynamics of key muscles when running at 5 m/s. (a) The average metabolic power consumed by the three muscles whose energy consumption decreased the most (vasti, iliopsoas, and gluteus maximus) and by the rectus femoris, whose energy consumption decreased marginally. (b) Mean hip adduction (top), hip rotation, hip flexion, and knee extension (bottom) moments are shown for the right leg when unassisted (dashed lines) and when assisted by an ideal hip flexion/extension actuator (solid lines), averaged across 10 subjects; the mean hip actuator torque from Fig 3 is shown for reference (black). The recruitment of the rectus femoris increased to generate more of the necessary knee extension moment during stance, and the superfluous hip flexion moment it generated was neutralized by the ideal actuator. The ideal actuator also provided the hip extension moment originally generated by the gluteus maximus during stance, thereby reducing the co-contraction of the gluteus maximus and gluteus medius in the hip rotation degree of freedom.

When the ideal hip flexion/extension actuator was added to the model, substantial changes in activations occurred in muscles crossing the hip and/or knee (Fig 4). In contrast to the ankle and knee, each of which has only one degree of freedom in our simulations, the hip is modeled as a ball-and-socket joint with three (purely rotational) degrees of freedom. As observed when assisting the knee, the activation of the rectus femoris increased to take advantage of its relatively high force-generating capacity—though, in this case, the increase in activity occurred during stance and enabled a decrease in the activity of the vasti (vastus lateralis, vastus intermedius, and vastus medialis). The ideal actuator neutralized the resulting superfluous hip flexion moment generated by the rectus femoris (Fig 6(b)). The ideal actuator also provided the hip extension moment originally generated by the gluteus maximus during stance, which reduced the hip external rotation moment generated by the gluteus maximus and, consequently, the antagonistic internal rotation moment generated by the gluteus medius.

**Fig 7:**
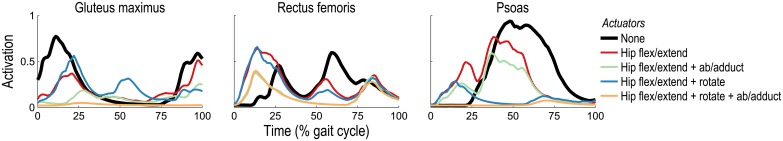
Activations of three representative muscles crossing the hip when running at 5 m/s with various hip assistive devices. Mean activations are shown for the gluteus maximus (left), rectus femoris (center), and psoas (right) on the right leg when running without assistance (black) and with four combinations of three hip actuators: flexion/extension only (red), flexion/extension with abduction/adduction (green), flexion/extension with internal/external rotation (blue), and all three actuators (orange). These results suggest that the gluteus maximus is particularly effective at generating a hip abduction moment because its activation decreased dramatically when the abduction/adduction actuator was added (e.g., compare the blue and orange curves). The rectus femoris does not appear to be especially favorable for generating a hip rotation moment because its activation was similar regardless of whether the internal/external rotation actuator was present (i.e., the red and blue curves are approximately equal, as are the green and orange curves).

A muscle’s relative effectiveness at generating a particular joint moment can be investigated by comparing activations when ideal actuators are added and removed. When assisting the ankle, for example, what remained of the original gastrocnemius medialis activation (see Fig 4) roughly reflects its activity attributable to generating a knee flexion moment during stance. Analyzing muscle activations when combinations of ideal actuators are added at the hip can help elucidate the contribution of each muscle to actuating each of the hip’s three degrees of freedom (Fig 7). For example, the gluteus maximus (which crosses only the hip) was inactive when all three hip degrees of freedom were assisted (orange curve in Fig 7) because any moment generated by the gluteus maximus could be generated by the ideal actuators at negligible cost. When the abduction/adduction actuator was removed, however, the activity of the gluteus maximus increased dramatically (blue curve), suggesting that this muscle is particularly effective at generating a hip abduction moment when running at 5 m/s. In contrast, the rectus femoris activation was nearly identical regardless of whether the internal/external rotation degree of freedom was assisted (e.g., compare the green and orange curves), confirming that the rectus femoris is not particularly effective at generating a hip rotation moment.

### Hypothesis Testing

We sought to test three hypotheses, the first being that a particular assistance location may be more effective at one speed than another, as reported when assisting the hip during running [31]. Our simulations support this hypothesis. For example, ideal ankle plantarflexion/dorsiflexion assistance was more effective when running at 2 m/s, decreasing average metabolic power by 26±4% (mean and standard deviation) at this speed but by only 19±4% when running at 5 m/s (Fig 1). In contrast, ideal hip flexion/extension assistance was more effective when running at 5 m/s, decreasing average metabolic power by 33±4% at this speed but by only 25±2% when running at 2 m/s. These results corroborate the findings of Sugar et al. [31], whose powered hip flexion/extension device reduced the metabolic cost of their “tall male” subject by 10.2% when running at 3.6 m/s (8 mph) but by only 8.0% when running at 2.7 m/s (6 mph). It may, therefore, be advantageous to design devices that can adjust assistance strategies with changing running speed, such as redistributing device power from the ankle to the hip as running speed increases.

Our second hypothesis was that the ideal assistive torque differs in magnitude and timing from the total joint moment generated during unassisted running, as suggested experimentally when assisting the ankle during walking [22]. When running at 5 m/s, this hypothesis appears to be true only for the ideal knee and hip actuators (Fig 3). Our simulations suggest that the ideal ankle assistance torque is very similar to the net joint moment. In contrast, the device of Collins et al. [25] generates a moment similar to that produced during unassisted walking but with lower magnitude, and Malcolm et al. [22] reported the greatest reduction in metabolic cost when their device began generating a plantarflexion moment significantly later into the walking gait cycle than the plantarflexor muscles. The difference in ankle assistance torques between these experiments and our simulations may be due to a fundamental difference between walking and running. In walking, the ankle plantarflexion moment generated during push-off coincides with a net flexion moment generated at the knee [54]. If assisting only the ankle, the gastrocnemii may still be recruited to generate the necessary knee flexion moment, whereupon an ankle plantarflexion moment would also be generated (reducing the demand on the assistive device). In running, however, the ankle plantarflexion moment generated during push-off coincides with a net knee extension moment (Fig 3). Because no muscles generate both ankle plantarflexion and knee extension moments, generating a knee extension moment using muscles has no potential to contribute to the required ankle plantarflexion moment. Other factors, including nonideal effects ignored in our study, may also explain differences between results from experiments and our simulations (see the Study Limitations section, below).

We note two additional observations related to the ideal assistive torques (Fig 3). First, our results for the ideal knee and hip actuators suggest that applying the total joint moment generated during unassisted running (or scaled versions thereof [29]) may not lead to the greatest reduction in metabolic cost. If each muscle generated a moment about exactly one degree of freedom, then the ideal assistive device would simply apply the total joint moment generated by the muscles when unassisted (minus passive muscle forces, which would be present even with zero activation); however, assisting biarticular muscles and muscles that cross joints with multiple degrees of freedom can affect moments generated elsewhere in the limb. Secondly, although the ideal hip flexion/extension actuator reduced average metabolic power more than the ankle or knee actuators did when running at 5 m/s, the standard deviation of the actuator torque was substantially greater. A larger standard deviation across subjects may correspond to greater difficulty in developing a single device that would accommodate different running styles. Of particular concern would be a device that mistimes a zero-crossing, generating a flexion torque when an extension torque would be desired, for example.

Our third hypothesis was that a device can decrease activity in muscles that do not cross the assisted joint, as observed when assisting the hip during walking [32]. Our simulations support this hypothesis. When assisting the knee, for example, the activity of the psoas (a hip flexor) decreased during early swing; when assisting the hip, the activity of the vastus lateralis (a knee extensor) decreased substantially during stance (Fig 4). Both effects occurred to take advantage of the relatively high force-generating capacity of the rectus femoris at these times. When assisting the knee, the increased hip flexion moment generated by the rectus femoris allowed the activity of the iliopsoas to decrease while the increased knee extension moment generated by the rectus femoris was neutralized by the ideal actuator (Fig 5(b)). When assisting the hip, however, it was the increased knee extension moment generated by the rectus femoris that was exploited, allowing the activity of the vasti to decrease, while the increased hip flexion moment was neutralized by the ideal actuator (Fig 6(b)). Note the substantial reduction in average metabolic power attributable to the knee extensors when the hip flexion/extension actuator was used (Fig 2).

### Summary of Observations

The metabolic power consumed by a muscle is highly dependent on its activity. Four general observations summarize the changes in muscle activity when assistance was added (Figs 4 and 7), and could be used to form hypotheses for future experimental studies:

1. Activation decreased dramatically when assisting all degrees of freedom actuated by a muscle (e.g., soleus and tibialis anterior when assisting the ankle; biceps femoris short head and vastus lateralis when assisting the knee; gluteus maximus and psoas when assisting all three degrees of freedom at the hip).
2. Activation can decrease when assisting some degrees of freedom actuated by a muscle (e.g., gastrocnemius medialis during stance when assisting the ankle; gluteus maximus during stance when assisting hip flexion/extension).
3. Activation can increase when assisting some degrees of freedom actuated by a muscle (e.g., rectus femoris during swing when assisting the knee and during stance when assisting hip flexion/extension).
4. Activation can decrease when assisting degrees of freedom not actuated by a muscle (e.g., vastus lateralis when assisting hip flexion/extension; psoas during swing when assisting the knee).

### Study Limitations

There are several limitations of this study. First, we generated muscle-driven simulations using the Computed Muscle Control (CMC) algorithm, which solves the muscle redundancy problem by minimizing the sum of squared instantaneous muscle activations. This objective produces realistic kinematics in predictive simulations of running [55] and results in realistic muscle recruitment patterns in CMC simulations of walking [56], running [42, 47], and other activities. Nevertheless, other factors likely contribute to determining muscle activity in assisted running, such as the amount of time a subject has spent wearing a device [38, 39]. We also ignored a subject’s efforts to maintain balance, avoid injury and fatigue, and minimize cost of transport. Furthermore, the stated objective of adding the assistive devices was to reduce metabolic cost, which was minimized only indirectly by minimizing muscle activity (Eq (1)). Finally, we note two specific weaknesses of the CMC algorithm: muscle activations are always at least 0.02, and the globally optimal solution to the minimization problem was not always found by the optimizer in our simulations. In particular, some activity remained in the soleus when assisting the ankle, in the vastus lateralis when assisting the knee, and in the psoas when assisting all three degrees of freedom at the hip (Figs 4 and 7); in all three cases, a lower objective function value (Eq (1)) could be achieved by generating these muscle moments with the assistive devices instead. Of these three muscles, the vastus lateralis had the largest activation during assistance, which translated into a relatively small amount of metabolic power (Fig 5(a)).

A second limitation of this study is our assumption that the kinematics and ground reaction forces observed experimentally during unassisted running would remain unchanged when assistance was added. Predictive simulations that discover kinematics and ground reaction forces could be used to investigate the validity of this assumption. Some evidence suggests that joint kinematics may not change substantially when assisted [25, 33] or may change initially and then gradually return to normal as the subject adapts to the device [38]; however, some studies report relatively large changes in kinematics as well [32, 57–59]. Nevertheless, changes in kinematics may not necessarily translate into substantial changes in metabolic cost [60]. The kinematic changes observed experimentally are likely a consequence of both the assistive forces applied to the body and the mass of the device—particularly if a substantial amount of mass is added to the foot [24].

A third limitation is that we modeled the assistive devices as ideal torque actuators—that is, the devices were massless, they could generate arbitrarily large torques instantaneously and with precise timing, and the penalty incurred for generating these torques was negligible. In practice, adding mass to the leg will increase energy expenditure during running. Compliant attachments of a physical device to the body [36] and limits on the magnitude and rate of the assistive torque could also reduce the effectiveness of an assistance strategy. These practical considerations may begin to explain why the reductions in metabolic cost we observed with the hip flexion/extension actuator (25–33%) exceeded the reductions in metabolic cost reported by Sugar et al. [31] for their powered hip flexion/extension device when running at similar speeds (8–10%).

A fourth limitation is that we did not model muscle fatigue, which may affect muscle recruitment strategies. For example, when the ideal hip flexion/extension actuator was added to the model, the activity of the rectus femoris increased during stance and enabled a decrease in the activity of the vasti (Fig 4). At this point in the gait cycle, however, the rectus femoris was lengthening while generating large forces and would, therefore, be susceptible to fatigue [61]. We also note that the rectus femoris is comprised of primarily fast-twitch muscle fibers [62, 63], which are more prone to fatigue than slow-twitch fibers [64]. Nevertheless, it may be possible to design a device that has the same effect as increasing the activity of the rectus femoris.

## Conclusions and Future Work

In this work, we used simulation to predict and gain insight into the biomechanical and energetic effects of assisted running, and to demonstrate the potential for simulation to complement experimental approaches to device design. We modeled several hypothetical assistive devices as ideal motors, predicted the optimal torque profiles and consequent reductions in metabolic cost, and sought explanations for the observed changes in muscle activity. We observed expected decreases in the activations of muscles crossing assisted joints, but also observed decreases in the activations of muscles crossing *unassisted* joints as well as *increases* in the activations of muscles with relatively high force-generating capacities. These adaptations in muscle coordination were observed when assisting single joints in ideal scenarios; practical devices assisting multiple joints simultaneously may lead to more complicated effects. It is, therefore, essential to incorporate biomechanical analysis into the assistive device design process.

By ignoring device mass and other practical factors, we avoided confounding the beneficial effects of adding assistance with the detrimental side effects often encountered experimentally; however, our simulations could also be used to investigate factors we did not consider in our study. For example, our simulations could be augmented with hypothetical device masses to study how the location and quantity of added mass affects energy expenditure during assisted running, while still avoiding the myriad experimental challenges facing device designers. Our approach could also be extended to investigate other practical effects, such as actuator limitations and muscle fatigue, and to study specific device designs. Validating these simulations and improving the available software tools are essential, and we hope that experimentalists will adopt and advance simulation-based assistive device design. The models, simulations, and software used in this study are freely available at simtk.org.
